# Epidemiological study of phylogenetic transmission clusters in a local HIV-1 epidemic reveals distinct differences between subtype B and non-B infections

**DOI:** 10.1186/1471-2334-10-262

**Published:** 2010-09-07

**Authors:** Kristen Chalmet, Delfien Staelens, Stijn Blot, Sylvie Dinakis, Jolanda Pelgrom, Jean Plum, Dirk Vogelaers, Linos Vandekerckhove, Chris Verhofstede

**Affiliations:** 1 AIDS Reference Laboratory, Ghent University, De Pintelaan 185-Blok A, B-9000 Gent, Belgium; 2 AIDS Reference Center, Ghent University Hospital, De Pintelaan 185-1P2, B-9000 Gent, Belgium

## Abstract

**Background:**

The number of HIV-1 infected individuals in the Western world continues to rise. More in-depth understanding of regional HIV-1 epidemics is necessary for the optimal design and adequate use of future prevention strategies. The use of a combination of phylogenetic analysis of HIV sequences, with data on patients' demographics, infection route, clinical information and laboratory results, will allow a better characterization of individuals responsible for local transmission.

**Methods:**

Baseline HIV-1 *pol *sequences, obtained through routine drug-resistance testing, from 506 patients, newly diagnosed between 2001 and 2009, were used to construct phylogenetic trees and identify transmission-clusters. Patients' demographics, laboratory and clinical data, were retrieved anonymously. Statistical analysis was performed to identify subtype-specific and transmission-cluster-specific characteristics.

**Results:**

Multivariate analysis showed significant differences between the 59.7% of individuals with subtype B infection and the 40.3% non-B infected individuals, with regard to route of transmission, origin, infection with *Chlamydia *(p = 0.01) and infection with Hepatitis C virus (p = 0.017). More and larger transmission-clusters were identified among the subtype B infections (p < 0.001). Overall, in multivariate analysis, clustering was significantly associated with Caucasian origin, infection through homosexual contact and younger age (all p < 0.001). Bivariate analysis additionally showed a correlation between clustering and syphilis (p < 0.001), higher CD4 counts (p = 0.002), *Chlamydia *infection (p = 0.013) and primary HIV (p = 0.017).

**Conclusions:**

Combination of phylogenetics with demographic information, laboratory and clinical data, revealed that HIV-1 subtype B infected Caucasian men-who-have-sex-with-men with high prevalence of sexually transmitted diseases, account for the majority of local HIV-transmissions. This finding elucidates observed epidemiological trends through molecular analysis, and justifies sustained focus in prevention on this high risk group.

## Background

Despite prevention campaigns and easy access to highly active antiretroviral therapy (HAART), the number of new HIV diagnoses in Western Europe continues to increase, with unprotected sex between men being reported as the main mode of transmission [1-3]. The design of prevention measures calls for a thorough understanding of the HIV epidemiology. As such, reconstruction of transmission networks based on interview data can provide valuable insights in the spread of the virus [4-6]. For several years now, international guidelines recommend baseline testing for drug resistance in all HIV-1 infected patients [7]. This has led to a substantial increase in the availability of viral sequence data and has permitted a new approach to study the HIV epidemiology [8]. More specifically, phylogenetic analysis allows the identification of mutual characteristics of so-called clusters, i.e. specific groups of patients in which multiple transmissions of HIV-1 have taken place. Studies using phylogenetics based on the *pol *gene of HIV were performed throughout the world to map local HIV epidemics in correlation with transmission pathway, drug resistance, risk behaviour and cluster size. Some focussed on the contribution of primary infection to onward transmission [9], while others investigated the transmission of drug resistant viruses [10-12] or concentrated on specific populations [13,14]. All these studies focus on the predominant subtype or the most predominant route of transmission, with little information on the other circulating subtypes or transmission routes. Although the presence of other sexually transmitted infections (STI) at the time of HIV infection has been associated with an increased risk of HIV infection [15] and a correlation between an increased incidence of syphilis infection and HIV has been reported [16], studies that link the presence of sexually transmitted infections with HIV transmission and the incidence of transmission clusters are rare.

The aim of this study was to evaluate whether phylogenetic analysis of data gathered through routine resistance testing procedures can supplement epidemiological data and allow a more detailed description of local HIV epidemics. The target population consists of HIV infected patients followed in a local AIDS Referral Centre in Belgium over 9 years (between January 2001 and March 2009). The selected cohort of patients is highly diverse with regard to ethnicity, transmission mode and HIV-subtype. By correlating the presence of transmission networks as established by the genetic relationship of the viruses, with information on demographics, transmission mode, CD4 counts, the presence of drug resistant virus and the presence of co-infections like hepatitis B (HBV) and C virus (HCV), syphilis and *Chlamydia *we wanted to obtain a better insight in the dynamics of the infection in this specific geographical area.

## Methods

### Study subjects

Between January 2001 and March 2009, 699 new patients were registered in the AIDS Reference Centre (ARC) in Ghent, Belgium. From this group, patients were retrospectively selected according to the following criteria: the patient was newly registered in the centre without documented previous follow-up elsewhere in the country, a plasma sample or plasma-derived RNA *pol *sequence was available within 1 year of initial presentation to the ARC, and the patient was treatment-naïve at the time of sampling. Patients signed an informed consent form for participation and the project was approved by the Ethics Committee of the institution. Patient information was collected anonymously from clinical files: HIV transmission route, gender, age, origin as well as CD4^+ ^T cell counts at time of diagnosis. Past or present HBV, HCV, *Chlamydia *and syphilis infection was considered based on the finding of HBV core antibodies (IgG + IgM), HCV RNA, a positive serology (IgG + IgM antibodies) and a positive TPPA test, respectively. Patients were considered in primary HIV infection phase (PHI) when a negative HIV screening result was available within one year of the first positive HIV test.

Of the patients with a subtype B infection, 94% are Caucasians (89% of Belgian and 5% of non-Belgian origin). The remaining 6% are from various parts of the world. Of the patients with a non-B infection, 59% is of African origin, 28% is Belgian and 5% is of a non-Belgian Caucasian origin. The remaining 8% is from various parts of the world.

### Samples

EDTA blood samples were collected regularly. The plasma fraction was stored at -80°C. Phylogenetic analysis was performed on plasma derived RNA sequences of samples collected within one year of the initial presentation in the clinic.

### RNA extraction, amplification and sequencing

Full length protease and partially reverse transcriptase sequences were amplified and sequenced from viral RNA extracted from plasma, as described previously [17]. After purification, sequencing products were analysed on the ABI 3130XL Genetic Analyser (Applied Biosystems Incorporated, Foster City, CA). Proofreading was performed with the IDNS software package (Smartgene, Zug, Switzerland).

### Phylogenetic analysis

Nucleotide sequences were assembled using the BioEdit package [18]. Sequences were aligned using Clustal W with manual correction [19]. Nucleotide gaps were assigned after amino acid conversion to maintain translation integrity, and gapped positions were removed. The data was split up in a subtype B and non-B group for computational reasons. For every M-group subtype, at least one reference sequence, retrieved from the Los Alamos Database, was added. To root the trees, the M-group consensus sequence (Los Alamos), was used [20]. The best fitting nucleotide-substitution model was a general time reversible model of nucleotide-substitution with a proportion of invariant sites (ι) and gamma distribution of rates (Γ), selected according to the Aikaike Information Criterium (AIC) using MrModeltest [21]. Maximum likelihood estimated distances according to the chosen model were used to construct neighbor joining phylogenetic trees. Bootstrap analysis was performed using the above mentioned conditions on 1000 replicates. All neighbor joining phylogenetic trees were reconstructed in PAUP* v4.0b10 [22]. Bayesian phylogenetic trees were constructed using MrBayes [23,24], under the same model conditions as described above with a sample frequency of 1000. Due to the size of the dataset and the limited computer capacity, analyses were run until the average standard deviation of split frequencies decreased below 0.05. At termination of the Bayesian analysis, parameters and trees were summarized with a burnin of 25%. The generation vs. log probability plots were checked for adequate sampling and potential scale reduction factors for convergence. Tree diagrams were plotted with Dendroscope [25]. Bias introduced by convergent evolution due to the presence of drug resistant mutations was avoided by repeating the analysis after removal of codons associated with transmitted drug resistance. The topology of the trees and identification of clustering remained unchanged (data not shown). The standardized list of mutations for surveillance of transmitted drug resistance (DRM) established by the World Health organization was used to identify resistance mutations [26].

### Identification of clusters

The initial identification of transmission clusters was based on the NJ trees topology. All clusters of 3 or more patients, with a bootstrap value higher than 90, were selected. Transmission pairs were excluded since they do not necessarily represent sources of continuous onward transmission. Clusters whose average genetic distance exceeded 0.015 were individually checked to evaluate whether the higher divergence of the sequences could be due to transmission events spanning several years and to avoid the inclusion of clusters of rare non-B subtypes. Subsequently a more robust method, Bayesian inference, was used to verify the clusters. Only clusters with a Bayesian posterior probability = 1 were considered as transmission clusters and selected for further analysis.

### Statistical analyses

Groups were compared using the chi-squared test for categorical variables and the Mann-Whitney U nonparametric test for continuous variables. In order to select the best significant predictive set of variables, a binary logistic regression was performed using a stepwise (enter method) procedure [27]. Hereby variables with a moderate relationship (p ≤ 0.2) with the dependent variable were included in the model, whereas a threshold of p > 0.05 was used for the stepwise elimination of potential risk factors. Goodness of fit of the final models was assessed by the Hosmer-Lemeshow test. The level of significance was set at p ≤ 0.05. All data was analyzed using SPSS 17.0 (SPSS Inc., Chicago, IL).

## Results

### Subtype-specific characteristics

Of the 570 patients that met the inclusion criteria of the study, protease and reverse transcriptase sequencing was attempted on 519. Drop outs were due to lack of patient information (n = 34) or a viral load that was too low for sequencing (< 200 copies/ml) (n = 17). The whole protease gene (297 nucleotides) and 568 nucleotides of the reverse transcriptase gene were successfully amplified and sequenced for 506 (97.5%) of the remaining 519 individuals. The majority of the patients was infected with a subtype B virus (59.7% vs. 40.3% non-B infections). Within the 17 identified different non-B subtypes, CRF02_AG (10.9%), CRF01_AE (9.7%) and C (6.1%) were the most predominant. Infections with the subtypes A1, F1, G, CRF06_cpx, CRF22_01A1, D, CRF12_BF, A2, F2, H, CRF03_AB, CRF09_cpx, CRF05_DF and CRF18_cpx were detected in <5% of the patients. For 2.8% of the patients, subtyping was inconclusive. A comparison of demographic and laboratory and clinical data of patients infected with subtype B and non-B strains is shown in table 1. Univariate analysis revealed major differences with regard to route of transmission, gender distribution, origin and the presence of syphilis and *Chlamydia *infection. Multivariate analysis showed an independent association between subtype B infection and homosexual transmission, Caucasian origin, Chlamydia and HCV infection.

**Table 1:** Comparison of characteristics of patients infected with a subtype B virus and patients infected with a virus of the non-B subtypes.

	Subtype B	Non-B subtypes		Multivariate Bin. Log. Regression		
	Count (%)	Count (%)	p-value	ODDS ratio	95% CI	p-value
**# Patients (n = 506)**	302 (59.7%)	204 (40.3%)				

**Homosexual transmission**	229/271 (84.5%)	13/137 (9.5%)	< 0.001	33.1	14.5 - 75.6	< 0.001
**HBV^+^**	106/296 (35.8%)	82/195 (42.1%)	0.164			-
**HCV^+^**	26/295 (8.8%)	8/197 (4.1%)	0.042	9.1	1.5 - 56.2	0.017
**Syphilis^+^**	119/300 (39.7%)	21/200 (10.5%)	< 0.001			-
**Chlamydia^+^**	106/259 (40.9%)	21/126 (16.7%)	< 0.001	3.6	1.4 - 9.6	0.01
**Gender (male)**	279/302 (92.4%)	94/204 (46.1%)	< 0.001			-
**Caucasian origin**	285/299 (95.3%)	68/202 (33.7%)	< 0.001	21.0.	5.3 - 82.5	< 0.001
**DRM**	25/302 (8.3%)	8/204 (3.9%)	0.052			-
**PHI**	60/275 (21.8%)	12/189 (6.3%)	0.021			-

	**Median (IQR)**	**Median (IQR)**	**p-value**			

**CD4^+ ^T cells (cells/μl)**	420 (284-592)	330 (182-516)	< 0.001			-
**Age (years)**	38.5 (32-44)	36 (30-36)	0.018			-

Values are given as counts per number of patients for which data was available. For the multivariate analysis, odds ratios are given with regard to Subtype B infection. The p-value for the Hosmer-Lemeshow test for the multivariate model is 0.528.

HBV, Hepatitis B virus; HCV, Hepatitis C virus; DRM, Drug resistant mutations; PHI, Primary HIV infection; IQR, interquartile range; CI, confidence interval.

### Transmission clusters

**Figure 1:**
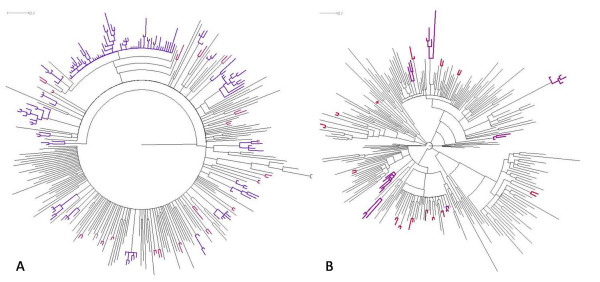
**Bayesian phylogenetic tree**. Panel A: Subtype B infections; Panel B: Non-B Subtype infections. Transmission clusters are indicated in purple, transmission pairs in red.

Twenty six transmission clusters and 35 transmission pairs were identified. For one small cluster, all 3 patients that formed the cluster were registered in the clinic during the same year. Twelve clusters included individuals who first consulted over a time span of 2 to 4 years, the remaining thirteen were clusters including patients whose first presentation covered 5 years or more. In the non-B subtype infections, 7 clusters were identified. The mean number of patients per cluster was 3.71 (range 3 to 6). Amongst the subtype B infections, 19 clusters were detected, one large cluster (comprising 57 patients) and 18 smaller clusters (mean number of patients: 4.78; range 3 to 10). Overall, 143 (47.4%) of the 302 subtype B infected patients belong to a transmission cluster versus 26 (12.7%) of the 204 non-B infected patients (p < 0.001). Phylogenetic trees based on the viral sequences of the subtype B infected and the non-B infected patients can be seen in figure 1. An overview of the characteristics of clustered and non-clustered patients, irrespective of the subtype, is given in table 2. Being part of a transmission cluster was significantly associated with harboring a subtype B virus, infection through homosexual contact, male gender, Caucasian origin, and syphilis. No association was observed between clustering and HBV or HCV infection and between clustering and the presence of drug resistant mutations (DRM). Multivariate analysis indicated homosexual transmission, Caucasian origin and lower age as the most potent predictors for being part of a transmission cluster.

**Table 2:** Comparison of characteristics of patients that are or are not part of a transmission cluster.

	∈ cluster	∉ cluster		Multivariate Bin. Log. Regression		
	Count	Count	p-value	ODDS ratio	95% CI	p-value
**# Patienten (n = 506)**	169 (33.4%)	337 (66.6%)				

**Subtype B**	143/169 (84.6%)	159/337 (47.2%)	< 0.001			-
**Homosexual transmission**	125/150 (83.3%)	117/258 (45.3%)	< 0.001	3.1	1.8 - 5.4	< 0.001
**HBV^+^**	56/167 (33.5%)	132/324 (40.7%)	0.12			-
**HCV^+^**	14/167 (8.4%)	20/325 (6.2%)	0.356			
**Syphilis^+^**	68/169 (40.2%)	72/331 (21.8%)	< 0.001			-
**Chlamydia^+^**	61/151 (40.4%)	66/234 (28.2%)	0.013			-
**Gender (male)**	154/169 (91.1%)	219/337 (64.4%)	< 0.001			-
**Caucasian origin**	158/166 (95.2%)	195/335 (58.2%)	< 0.001	14.5	4.2 - 49.9	< 0.001
**DRM**	12/169 (7.1%)	21/337 (6.2%)	0,709			
**PHI**	34/162 (21.0%)	38/302 (12.6%)	0.017			-

	**Median (IQR)**	**Median (IQR)**	**p-value**			

**CD4^+ ^T cells (cells/μl)**	419.5 (311-580)	351 (192-550)	0.002			-
**Age (years)**	36 (31-42)	38 (32-45)	0.067	0.9	0.92 - 0.97	< 0.001

Values are given as counts per number of patients of which data was available. For the multivariate analysis, odds ratios are given with regard to being a member of a cluster (∈ cluster). The p-value for the Hosmer-Lemeshow test for the multivariate model is 0.876.

HBV, Hepatitis B Virus; HCV, Hepatitis C Virus; DRM, Drug Resistant Mutations; PHI, Primary HIV Infection; IQR, interquartile range; CI, confidence interval.

To avoid interpretation bias resulting from the inclusion of one large cluster of 57 males, all of Caucasian origin and infected through homosexual contact with a subtype B virus (further referred to as the MSM-cluster), the statistical analyses were repeated after exclusion of these 57 individuals. The significant associations of clustering with subtype B infection (p < 0.001), homosexual transmission (p < 0.001), male gender (p < 0.001), Caucasian origin (p < 0.001) and syphilis (from p < 0.001 to p = 0.003) remained. On the other hand, clustering associated more weakly with *Chlamydia *infection (from p = 0.013 to p = 0.097), CD4 cell counts (from p = 0.002 to p = 0.022) and presentation during acute infection (from p = 0.017 to p = 0.120).

### Characteristics of the MSM population

Information of the transmission route was available for 408 of the 506 patients. Of these, 242 (59.3%) reported transmission through homosexual contact, 154 (37.7%) through heterosexual contact and 7 (1.7%) through intravenous drug use (IVD). Ten patients reported other infection routes (bisexual contact, blood transfusion, needle accident,...). IVD represents a marginal proportion of the transmissions in the total population.

Multivariate analysis assigned a significant predictive value of homosexual transmission to infection with a subtype B virus (p < 0.001), Caucasian origin (p = 0.002), past or present syphilis infection (p < 0.001), being part of a transmission cluster (p = 0.004), past or present HBV (p = 0.012), and, with borderline significance, infection with a drug resistant virus (p = 0.05) (Table 3). Univariate analysis additionally showed strong association with Chlamydia infection (p < 0.001), acute stage of infection at initial presentation (p < 0.001) and higher CD4^+ ^T cell count (p = 0.001). The same associations were found after removal of the 57 patients of the MSM-cluster.

**Table 3:** Comparison of the patients being infected through homosexual transmission with patients infected through other routes.

	MSM	Other than MSM		Multivariate Bin. Log. Regression		
	Count (%)	Count (%)	p-value	ODDS ratio	95% CI	p-value
**# Patienten (n = 408)**	242 (59.3%)	166 (40.7%)				

**Subtype B**	229/242 (94.6%)	42/166 (25.3%)	< 0.001	23.1	10.2-52.3	< 0.001
**Cluster**	125/242 (51.7%)	25/166 (15.1%)	< 0.001	2.8	1.4-5.6	0.004
**HBV^+^**	88/236 (37.3%)	49/159 (30.8%)	0.185	2.7	1.2-5.7	0.012
**HCV^+^**	16/237 (6.6%)	14/160 (8.8%)	0.46			
**Syphilis+**	108/242 (44.6%)	13/162 (8.0%)	< 0.001	7.1	2.9-17.3	< 0.001
**Chlamydia^+^**	90/220 (40.9%)	18/97 (18.6%)	< 0.001			-
**Caucasian origin**	233/240 (97.1%)	88/164 (53.7%)	< 0.001	6.7	2.0-22.1	0.002
**DRM**	22/242 (9.1%)	4/166 (2.4%)	0.007	6.1	1-36.6	0.05
**PHI**	54/226 (23.9%)	14/150 (9.3%)	< 0.001			-

	**Median (IQR)**	**Median (IQR)**	**p-value**			

**CD4 (cells/μl)**	426 (299-597)	341 (181-528)	0.001			-
**Age (years)**	38 (32-44)	37 (32-43)	0.351			

Values are given as counts per number of patients of which data was available. For the multivariate analysis, odds ratios are given with regard to MSM. The p-value for the Hosmer-Lemeshow test for the multivariate model is 0.625.

MSM, men who have sex with men; HBV, Hepatitis B Virus; HCV, Hepatitis C Virus; DRM, Drug Resistant Mutations; PHI, Primary HIV Infection; IQR, interquartile range; CI, confidence interval.

### Presence and transmission of primary drug resistance

Baseline drug resistance mutations (DRM) were detected in 33 patients (6.5%). NRTI mutations only were found in 19, NNRTI mutations in 6, PI mutations in 4, a combination of NRTI and NNRTI mutations in 2 and a combination of NRTI and PI mutations in 2. A high prevalence of revertant mutations at amino acid 215 of reverse transcriptase (RT) was observed, with 18 of the 23 patients with NRTI mutations having either the amino acid D, E, C or S at that position instead of the wild type T or the drug resistant Y or F. A significantly higher frequency of DRM was observed in patients infected through MSM (9.1% vs. 2.4%; p = 0.007). Also an association between DRM and infection with subtype B virus was noticed but the support for statistical significance was weak (8.3% in Subtype B vs. 3.9% in Non-B subtypes; p = 0.052; see table 1). No association between the presence of DRM and presentation during primary infection was seen. Fifteen of the 33 patients with DRM were localized on separate branches of the phylogenetic tree, 18 were part of transmission pairs or transmission clusters. In one cluster, the 215 D revertant was detected in all 5 patients. In another cluster of 4 patients, all were infected with a virus carrying the 215E revertant. One patient of the latter cluster additionally had a 41L. In 2 transmission pairs, both individuals carried the same NNRTI mutation (190A and 106A, respectively). In 5 patients, mixtures of wild type and drug resistant virus were observed. All were part of a transmission pair or a transmission cluster of 3-5 individuals but were the only members of the cluster with DRM.

## Discussion

For 506 patients, newly diagnosed and consulting a university HIV clinic between 2001 and 2009, data on patient demographics, infection risk and laboratory and clinical parameters, were supplemented with information obtained from phylogenetic analysis of the HIV-1 sequences. The results revealed an epidemic characterized by high heterogeneity in subtypes and high prevalence of non-B infections (40.3%). This heterogeneity and high non-B prevalence has been a characteristic of the HIV epidemic in Belgium since the beginning [28,29]. From the results of our study it appears that in our local cohort, even after years of co-existence of different subtypes, patients infected with subtype B viruses and non-B viruses still represent highly distinct populations with regard to route of transmission (84.5% homosexual transmission for subtype B vs. 9.5% for non-B; p < 0.001), origin (95.3% Caucasian origin for subtype B infection vs. 33.7% for non-B; p < 0.001), gender (92.4% males for subtype B infection vs. 46.1% in non-B subtypes) and detection of other STI infections (Syphilis and/or *Chlamydia *infection 59.9% for Subtype B infection vs. 28.1% for non-B subtypes; p < 0.001).

In addition, more and larger transmission clusters were defined in subtype B infected patients compared to the non-B infections (19 clusters comprising 143 of the 302 patients infected with subtype B viruses and 7 clusters comprising 26 of the 204 patients with non-B infections; p < 0.001). These figures suggest that local onward transmission of subtype B virus contributes to an important extent to the epidemic. As 95.3% of the subtype B infected individuals are Caucasian and most are local citizens, the chance that the source patients or sexual partners are followed in the same hospital is higher than for the non-B infected patients of which 66% are foreigners and an important part is infected in the country of origin. This might in part explain the observations. Gifford et al. also showed that the majority of non-B infections in the UK in 2007 reflects separate introductions through travel and migration [30].

Importantly, this study shows the clear association between phylogenetic clustering, homosexual HIV transmission and infection with other STI. A correlation between being infected with closely related viruses, sexual risk and sexually transmitted infections has been shown before [15] in a study that was performed on a mainly Caucasian and almost exclusively MSM population (91% MSM). Our study included female and male patients of several origins, infected with a variety of HIV subtypes and through several transmission routes.

The observation of one very large transmission cluster of 57 MSM, infected with genetically very similar viruses (mean genetic distance: 0.0108), is striking and alarming. The cluster contains patients diagnosed over the whole 9 year follow-up period, with 8 new inclusions in the last year. Members of this cluster are significantly younger than the rest of the population (0.022) and have more Chlamydia (p = 0.013) and syphilis infections (p < 0.001).

Even after exclusion of this one cluster the significant association between clustering, MSM infection and syphilis infection remains. Together, these observations indicate that high-risk taking MSM constitute the most important source of local onward HIV transmission in our region. These findings corroborate the results of national survey programs in Belgium, revealing a continuous increase in number of new infections that is attributable to a large extent to increasing incidences in the MSM population http://www.iph.fgov.be/.

Because of the retrospective nature of this study, only the available information on STI screening results could be used. Distinguishing between an infection from before or after the diagnosis of HIV-1 infection is often impossible. Prospective studies that check for ongoing STI's are necessary to further address the role of STI's in HIV-1 clustering.

Previous phylogenetic studies, that concentrated mainly on subtype B infections (Brenner et al., 2007: 90% Subtype B) [9] or on patients from Caucasian origin (Yerly et al., 2001: 94% Caucasians) [31] reported an association between clustering and primary HIV infection. This association was confirmed by our data although the statistical support was weak (p = 0.017). A more significant association between clustering and higher CD4 counts (p = 0.002) however, supports the relation between clustering and earlier infection stage. In contrast to the studies cited above, the stage of infection was unknown for most of the patients included in our study. It is possible that our selection criterion for PHI patients has created a bias toward the preferential inclusion of patients who present for regular screening. These individuals are most probably more aware of their risk taking behavior and the association seen between clustering and PHI might reflect an association between clustering and high risk behavior.

One of the major difficulties when running phylogenetic analysis on HIV-1 sequences in an attempt to identify clusters of onward transmission, is the lack of well defined criteria for cluster identification. Selection of clusters based on bootstrap values only, as done by some [31,32], implies a risk for the erroneous inclusion of subtype-specific clustering. On the other hand, bootstrap values can be misleadingly low in case of clusters with very short branch-lengths due to high similarity of the viruses [33]. Selection of clusters based on low genetic distance, as done by others [8,9,11,12,15,34] will restrict the inclusion to transmission events that occurred within a short timeframe. Inclusion of reference sequences and the use of robust methods can help identifying these problems. For our study, we opted for very strict criteria, based on bootstrap values and Bayesian probability and with an individual check of all clusters with a mean genetic distance >0.015. A drawback is that this might have caused an underestimation of the number of transmission clusters but it prevents the inclusion of clusters with a low chance of being attributable to local transmission events.

The presence of mutations possibly associated with a reduced sensitivity for antiretroviral drugs was observed in 6.5% of the patients analyzed. This figure is somewhat lower than the overall reported percentage for Belgium (9.5%) and the mean percentage for Western European countries (9%) [35,36]. The observed higher prevalence of DRM in subtype B than in non-B infections confirms the results of others [37]. Although it was not one of the major aims of this study to investigate the distribution of drug resistant virus in our population, the available information provided some interesting insights in the transmission of these DRM strains, as it demonstrates the frequent transmission of viruses containing 215 revertant mutants. These mutations evolve from 215 resistant mutations after primary infection with resistant strains and therefore are supposed to indicate the latent presence of resistant variants. Highly sensitive sequencing techniques however failed to identify these resistant variants in the majority of patients in whom the revertant mutation was detected [38]. These findings and our observations indicate that an important number of 215 revertants are the result of ongoing infection with revertant strains and that their presence will have no or only limited influence on the virologic response to antiretroviral medication.

## Conclusions

This study proves that a complementation of information on patients' demographics, transmission mode, viral and clinical parameters, with results obtained through phylogenetic analysis of sequences that are available through routine drug resistance screening programs, creates an added value to the understanding of local HIV epidemics. This deeper comprehension can in turn contribute to the design of well targeted prevention and control programs. We were able to show that patients infected with subtype B virus and patients infected with non-B virus represent significantly different populations. We clearly demonstrate that, despite the existence of prevention programs, easy available testing facilities and a supposedly broad public awareness of the infection and its possible routes of transmission, MSM still account for the majority of local onward HIV transmissions. Continuous efforts to sustain prevention programs targeting MSM are definitely needed, and the opening of a debate on the appropriateness of systematic treatment of MSM meeting some of the characteristics associated with a higher chance of being a transmitter, is indicated.

## Competing interests

The authors declare that they have no competing interests.

## Authors' contributions

KC designed and performed the experiments and the phylogenetic and statistical analysis; prepared the manuscript; DS performed and validated the sequencing reactions; SB guided the statistical analysis; SD collected the clinical data from patient files; JPelgrom and LV were responsible for patient inclusion and clinical follow-up; JPlum and DV gave support and conceptual advice for the design of the study; CV developed the concept and supervised the study at all stages. All authors discussed the results and commented on the manuscript and all read and approved the final manuscript.

## Pre-publication history

The pre-publication history for this paper can be accessed here:

http://www.biomedcentral.com/1471-2334/10/262/prepub
